# Observations on the Severe Dysentery, as It Existed on Board the Lord Duncan East Indiaman during a Voyage to Bengal, in 1802—4. In a Letter to John Hunter, M.D. F.R.S. Physician to the Hon. East India Company

**Published:** 1804-06-01

**Authors:** James Atkinson


Observations on the Severe Dysentery, *as it existed on board the Lord Duncan East Indiaman during a Voyage to Bengal, in* 1802—4. *In a Letter to* John Hunter, M. D. F. R. S. *Physician to the Hon. East India Company. By* James Atkinson.

Dear Sir,

NOTWITHSTANDING the multiplied labours of medical men for the elucidation of difficult and obscure points in the profession, and acknowledging with due admiration the vast improvements of late years, there is much left behind to exercise the sagacity, the talents, and discrimination of succeeding practitioners. They have not yet acquired that quickness of perception, and soundness of judgment, which must enable them fully to comprehend the phenomena of disease. They have, however, been indefatigable in their inquiries; and such exertions as tend to alleviate the anguish of disease, and to lessen the weight of human misery, must ever be regarded as noble and exemplary: for those feelings are surely the most honorable, which excite a man, independently of his professional obligations, to devote his time and abilities to the common interest; and the more dangerous or fatal the maladies are which we have to encounter, the more it requires of steady and unremitting zeal in the prosecution of our studies.

From the incommodious situation of the sick on board a ship, and their want of proper attendance, the medical practitioner labours under numberless disadvantages. Often his intentions are rendered useless by their neglecting the regulations prescribed; every auxiliary means is probably disregarded; and those who do recover from any malignant disorder, are generally indebted to the effects of of medicine alone! On entering the sick-birth, where a number of patients labour under a complication of diseases, the mind, filled with a serious emotion, must frequently advert to that awful scene, so well described by our immortal poet, where he emphatically enumerates the miseries which harrass mankind.

——— Pining atrophy,
Dropsies, and asthmas, and joint racking rheums;
Dire was the tossing, deep the groans; Despair
Tended the sick, busiest from couch to couch;
And over them, triumphant Death, his dart
Shook,—but delayed to strike!

The ingenious and profound publications of Hunter, Pringle, and Lind, may be supposed to have rendered any further remarks on the Dysentery superfluous, and obtrusive; but as long as diseases resist the operation of medicine, there can be no end to investigation. The object of these pages is to describe a practice in the treatment of this formidable and distressing disease, which I have found peculiarly successful. The facts adduced, occurred during a voyage to Bengal. The fatal cases, as well as those of favorable event, are faithfully mentioned, for this is an ingenuousness we owe to society in every scientific and medical inquiry.

The Lord Duncan* sailed from the Downs on the 14th of October, 1802, and arrived at the Cape of Good Hope on the 22d of December; during which time two seamen were attacked with dysentery; at the Cape one of them, who was advanced in life and very infirm, died. On the 24th of February, 1803, three hundred and sixty soldiers embarked for Madras. In the passage of about eight weeks, twenty had severe dysenteric complaints, one of whom died, and an infant child. Having wet, tempestuous weather off the Cape, the disease appeared epidemical. At Madras, and from thence to Diamond Harbour, a period of a month, eight had the dysentery. During the ship's stay in India, which was six months, a great number suffered severely under the same complaint, but only one died. Off Kedgeree and Saugor Island, in September and October, the remittent fever was very severe, and often fatal, the dysentery raging at the same time.† Homeward-

* The voyage was completed in eighteen months.

† The number of sick on board the Lord Duncan, during the six months in India, was 105, and only two died of disease, and one from an accident!

Ths ward bound, many slight cases occurred, and some in a violent degree. Calomel was the remedy always resorted to with advantage.

The carpenter, a middle aged man, reduced by almost continual intoxication to a tottering debility, being harrassed by quotidian ague and the dysentery, died as we left Bengal.

Excessive heat and cold are powers perhaps equally debilitating, but the alternation of heat with cold is more injurious than either. Heat succeeded by cold and moisture, is generally, I believe, one principal cause of the dysentery; and Dr. Cullen observes, that "it happens in the same circumstances and seasons which considerably affect the state of the bile in the human body. But as cholera is often without any dysenteric symptoms, and copious discharges of bile have been found to relieve the symptoms of dysentery, it is difficult to determine what connection this disease has with the state of the bile."

Does it not appear more than probable, that a deficiency rather than a redundancy of bile in the alimentary canal, is the cause of the dangerous symptoms of the dysentery? for from derangement in the functions of the liver, the bile, whose properties are known to be antiseptic, is prevented

---

This is a very small proportion considering the oppressive heat of the climate, and the various noxious exciting powers to which a seaman's life is continually exposed!

Windsails and fumigation, with sulphur and gunpowder, were constantly employed in the voyage outward. It may be observed that swabbing the beams and deck with common vinegar, though recommended in medical writings for ages past, is not attended with its boasted good effects. The volatile or spirituous part of it soon evaporates, and the consequent dampness, which is so extremely prejudicial to the healthy and much more to the diseased state, is sufficient confirmation that no salutary effect can result from its use. Even distillation (Lavoisier) does not deprive this acid of all its unnecessary water; for this purpose it may be exposed to a degree of cold from 19° to 23° of Fahrenheit; by this means the aqueous part becomes frozen and leaves the acid in a liquid state, considerably concentrated. If half the quantity of vinegar that is wasted in the manner above alluded to, was served out as a corrector of salted diet, to the ship's company, I am persuaded the scurvy would be much less prevalent and seldom so formidable in its symptoms and progress. A good allowance of this article and mustard, perhaps was the cause of an exemption from any scorbutic complaint in the outward bound voyage. I would also strenuously recommend fumigation with nitrous vapour. It is of superior efficacy in preventing and mitigating the violence of disease, and also much less expensive and unpleasant than the method above mentioned. It oxygenates the atmosphere more successfully, and its universal adoption in his majesty's navy, is the most forcible testimony that can be urged in favour of its usefulness.

prevented from passing through its proper and legitimate channel, and from this source the mucous excretions become so putrid and acrimonious. Obstruction in the common duct may be owing to spasm, torpor, or paralysis; and when there is pain over the pit of the stomach, which very frequently occurs, I suppose the seat of it to be the extremity of the bile duct, where it terminates in the duodenum.

In England, the dysentery or cholera occurs generally about autumn, and more particularly after a sultry summer, when bilious intermittent and remittent fevers prevail. It is seldom fatal, and when that does happen, no ulceration or tubercles in the intestines can be found;* nothing more than a gentle laxative, with proper regimen and cordials, being required to effectuate a cure.

There is perhaps some plausibility in the opinion, that the nature of the dysentery resembles the epidemic catarrh or influenza. The application of cold and moisture will certainly produce both diseases. The mucus membrane of the nostrils is similarly affected to the mucus membrane of the intestines; and to there being more extension of surface in the latter, may be ascribed the greater severity of the dysentery. In the catarrh, the mucous, from its ichorous quality, inflames and swells the lip; and inflammation of the inferior part of the rectum, will occasion the distressing tenesmus which is such an agonizing symptom of the dysentery. The afflux of humours in both, is of the same purulent kind. The liver too of those who died of *catarrhus contagiosus*, has been generally diseased;† and the same method of cure will nearly apply to them both. These circumstances strongly point out the analogy between the two diseases.

In the severe dysentery of hot climates, the liver is more or less affected, and hence the increased violence of every symptom. The eyes of every patient which I have seen, have been considerably tinged with yellow. The obstruction in the *ductus communis*, for bile has been said to be the natural purge of the intestines, and the inverted motions of the lacteals from debility, account for the white mucous evacuations, resembling chyle; and hence the salutary effect of mercury and opium in removing the offending cause, and promoting the healthy action of a set

---

* Vide Baillie's excellent book on Morbid Anatomy, p. 175.

† Darwin's Zoonomia.

set of vessels so important to the support of the system. According to this opinion, brisk cathartics, as they carry off so much of the fluid, elaborated by the process of digestion, are improper. But a Dr. Wade has written a book,* wherein he attacks with haughtiness and vehemence, all practice but that which he struggles to defend, viz. *purging!* Never man manifested more anxiety and groaning of spirit than he does in persuading his readers of the incontrovertible truth of his doctrine. Every medicinal power, though recommended by the consent of the highest characters, he contumaciously rejects, to give room for the universal purging despot, whose influence he wishes to extend over every form and modification of disease. This is in perfect harmony with the old system, which enjoins bleeding in hæmorrhages though in a nearly exhausted subject. *Cantilenam eandem canis.*

In a book called Observations on Hepatic diseases, the dysentery is attributed to a diseased liver. Though written in a curious style "with many holiday and lady terms," the arguments appear sufficiently conclusive. The fatal cases and the dissections are related; and in all, the liver or its appendages were found in a morbid state. Sir John Pringle describes four dissections of dysenteric patients. In the first, the liver was not apparently altered by disease, but the bile was thick, ropy, and of a dark hue. In the second, the liver was small and sound, but the gall-bladder was of an uncommon size and full of dark coloured bile, thin though somewhat curdled. An enlarged spleen. In the third, the liver was of an extraordinary bulk, weighing ten pounds, with an abscess. The gall-bladder was of an immoderate size, and full of thin black bile. In the fourth, the liver was in a sound state, the gall-bladder was empty of bile and contained only air. In all these cases, mortification of the large intestines had taken place, and a tenderness of all the viscera, the whole alimentary canal containing nothing but air.

From this affection of the liver, Sir, which has hitherto been overlooked by most practitioners, or only regarded as merely adventitious, I conceive the disease, both in the East Indies and everywhere else, to be so severe and dangerous. But this affection does not *alone* demand the necessity

---

* See Select Evidences, &c. &c. by John Peter Wade, M.D.

cessity of mercury* in the dysentery. Dissertations have been purposely written to recommend it even in low fevers, and its specific power in many infectious diseases, as syphilis, &c. warrants the opinion that contagion itself affecting the human system, may be deprived of its principal force by the prudent administration of this celebrated mineral.

It would be an important question to decide, Sir, whether the dysentery is, or is not contagious. The inquiry would involve an extensive discussion incompatible with the limits prescribed for this work, but a few observations may be necessary. The opinion that the dysentery arises from a specific contagion is certainly unfounded. In crowded ships the best opportunities present themselves of ascertaining this fact, and I never saw a case which could be referred with certainty to contagion. Pringle seems to doubt the sagacity of Sydenham and Willis, because they did not find that the dysentery was contagious. Sydenham's history of the epidemic, I believe to be very faithful and accurate; and no symptom or medical fact could have reasonably escaped the notice of such an eminent practical physician. Experience qualifies the proposition, that the same noxious exciting powers will act differently on different constitutions. If they induce diseases depending on debility, the indication of cure will be the same; as the system, whether affected with the dysentery, typhus fever, or other asthenic diseases, requires stimulant operation for the re-establishment of the healthy state. Leeuwenhoeck, the celebrated microscopical philosopher, and Linneus, discovered through their glasses, insects in the mucus of the

* When even mercury does not produce soreness and tenderness of the mouth, it is probably of no service. I remember seeing a young man of a weakly habit at Calcutta, under the care of a surgeon of some eminence, who had been ill of the severe dysentery twenty days, and during that time had used two drachms of ung. hydrarg. and about twelve grains of calomel every day. His mouth was not the least affected and the disease had not abated in its violence. In this case the calomel was not combined with any opiate or astringent, which accounts for the continuance of the flux. I afterwards heard, that on the fourth week of the disease, abscess of the liver had formed, and its suppurating internally, occasioned his death. The Herculean practice which has become so popular with respect to mercury, and which seems to be absolutely necessary to obviate the fatal tendency of diseases incident to tropical climates, has astonished many medical men who have visited the hospitals in India. And even private individuals, aware of its efficiency, have calomel constantly by them, which they take immediately on the approach of indisposition.

intestines of dysenteric patients; and it has been sagely conjectured, that floating in the atmosphere, they have produced the complaint. Consequently, the fæces not being immediately removed, have been supposed to be highly contagious. By increasing the malignity of the air we breathe, they will of course be injurious, but they will not produce the dysentery by a specific power. It would be well if medical men would attend more seriously to this question, and not have such an implicit dependance on the representation of others.

Along with the mucus the villous* coat of the intestines is frequently separated and discharged, as appears by its filamentous and organic structure. The same coat of the stomach is also often abraded. Thus the tunica nervosa becomes exposed and bare, and hence that pungent and scalding pain on swallowing solid food and even liquids in the severe stage of the dysentery.

Various reasons have been given, of so much pure blood being evacuated by stool in this disease. Hæmorrhagies of this kind generally proceed from deranged action of some part of the venous system. For when either direct or indirect debility is established, or the excitability nearly consumed by repeated intoxication, the veins cannot propel the blood forward to the heart with sufficient impetus, as is exemplified by the oozing of thin dark coloured blood, from different parts of the body, in the last stages of putrid fever and scurvy. This diminished energy, when there is an hepatic affection, may predominate in the vena portarum,† and hence the great quantity of blood which is frequently discharged in the severe dysentery. The symptom is not uncommon amongst those much reduced by an excessive indulgence in strong fermented liquors.

From my experience in marking the characteristic symptoms of the dysentery, the patient first complained of chilliness and lassitude, with dry febrile heat, and then griping pains, and frequent purging, attended with tenesmus. The pulse was small and quick, a suffusion of yellow soon appeared over the tunica albuginea, and the complexion a little jaundiced. The evacuations by stool at first, were of frothy mucus, and occasionally streaked with blood, but never of a scyballous appearance; and what is perhaps

* Vide Edinburgh Essays, vol. vi. p. 150.

† Dr. Saunders supposes the chronic inflammation of the liver to originate in the vena portarum; and a flux of blood downwards is an indication of a diseased liver.

perhaps more singular, there was no flatulence in the bowels. The griping and tenesmus became excessively severe. The calls to stool grew almost constant, but nothing was voided, except a little slimy tenacious mucus. Some had frequent prolapsus ani, violent dysuria, and lancinating pains over the pelvis and abdomen. There was an evident imbecility in the intellectual functions, with an incoherence of thought and expression. The pupil was contracted. A hiccup came on. Sometimes black fœtid matter was vomited, and the most excruciating symptoms supervened. The pulse became languid, and scarcely perceptible; the stools intolerably offensive, often nothing but blood; and the disease being left to itself, death soon closed the catastrophe.

I have thus drawn the progress of the Dysentery, as uninterrupted by the operation of medicine; but when early application is made for assistance, the dangerous symptoms are generally soon alleviated.

The sympathy between all the floating viscera of the abdomen is sufficiently proved by experience. When the intestinal canal is diseased, the contiguous membranes will become diseased also. The mesentery will inflame, suppurate, mortify, or become schirrous; and from induration of the glands, a wasting of the body will inevitably ensue, and a gradual decay of the energies of nature. On this principle we may account for that lingering state of the dysentery, when not even a glimmering of hope can be entertained of a favourable termination.

Respecting the cure, Dr. Mead says, "Semper primum sanguinem mittere expedit;" but the adoption of such practice is often pregnant with much mischief, and is always inadmissible in a hot climate. I never saw inflammatory symptoms so urgent as to require particular attention. On the contrary, the torpor of the brain, stomach, and other important viscera, indicate a penury rather than an exuberance of blood; and the rapid decay of all the vital powers, is the most pressing and dangerous enemy that we have to cope with. Emetics have been greatly recommended, and at the commencement they may be of use in evacuating and clearing the biliary ducts, but afterwards I conceive them to be very doubtful auxiliaries. The application of a blister on the abdomen is much more distressing to the patient, than any benefit I ever saw derived from it. Peruvian bark conjoined with opium was given with an intention of more certainly counteracting the dissolved state of the fluids, but the stomach seldom retained it. The addition of absorbents and aromatics were often also unsuccessful. Sir J. Pringle, apparently, thinks every purgative beneficial; but the ease obtained by purges is always momentary and fallacious. The temporary exhaustion produced by such medicines, gives deceitful quiet, which is constantly succeeded by still more violent pains than before. You, Sir, have also recommended purgatives in your valuable and interesting Observations on the Diseases of Jamaica; they may have done good, but I have not myself found their efficacy in the few cases that have come under my observation. The exhibition of enemas I had formerly found, by their mechanical irritation, only exasperated the symptoms, and more particularly when the rectum was supposed to be ulcerated. Warmth, in general, is of manifest advantage; fomenting the abdomen with warm vinegar, and afterwards the application of a few folds of flannel round the body, being of eminent service. The torpor and inverted motion of the stomach will be relieved by elixir of vitriol, opium, and the essential oils. The combination of opium and calomel, prevents the catharsis and nausea which would result from the use of calomel alone, and they form perhaps the noblest remedy we are yet acquainted with.*

We are even told by Dr. Killner† that the dysentery has been cured by a violent drubbing; which, though whimsical enough, is, I think, extremely probable, as the excitement occasioned by such a stimulus, will produce an accumulation of sensorial power, according to Dr. Darwin, and thus overcome the torpor and debility attendant on the disease. The learned votaries of Venus can explain similar curious phenomena. And it is a known fact that a diarrhœa will be checked or removed‡ by the stimulus of a flannel shirt worn next the skin.

The celebrated Sydenham had great confidence in the powers of laudanum, which he says is alone sufficient to perform a cure. About his time many were prejudiced against the frequent use of that invaluable medicine; but Sydenham found

* A small dose of Dover's powder, or antimonium tartarizatum, every night at bed-time, joined with the mercurial and opiate medicine, seems necessary to the cure.

† Vide Act. Acad. Naturæ Curiosorum, vol. iv. obs. 20. See also a case mentioned by Darwin of two patients in the Edinburgh Infirmary ill of the dysentery, who recovered from the disease, after being stimulated by anger and fighting.

‡ Zoonomia.

found out its virtues, and he thanks the Omnipotent for such a blessing, in a strain of enthusiastic devotion.

From this view of the subject and the medicines which have been recommended, the plain conclusion is, that the dysentery being a disease of the highest debility, chiefly predominant in the alimentary canal, those medicines which produce excitement over the system are only applicable; and particularly the following. Calomel with tartarized antimony and opium; cheerful emotions of the mind; a rich nutritious diet, and Port wine.

Having hastily premised these remarks, which are the result of careful observation, uninfluenced by any preconceived opinion, the succeeding Cases will illustrate the principle and practice more clearly.

### CASES.

1. Isaac Morn, armourer, of a robust habit, was seized with frequent purging of mucus and blood on the 21st of May. Six grains of calomel were prescribed for him, and an anodyne at night.

Second day of his illness. Has had acute pain in the lower part of the abdomen. Has great thirst, tongue dry and furred, with a small rapid pulse. Countenance yellow. The stools are still mucous and bloody, and very frequent. Take six grains of calomel joined with one grain of opium directly, and rub in ung. hydrag. ʒjß. on the region of the liver, twice a day.

Third day. Slight nausea; flushes of heat; pulse weak, tongue foul, and thirst urgent. Pain over the scrobiculus cordis. Has had but six stools the last twelve hours, principally of a greenish yellow, and without any streaks of blood. Take frequently of an acidulated drink and repeat the calomel cum opio three times a day, continuing the ointment.

Fourth day. Had considerable griping all day yesterday, and his stools were extremely offensive. The pulse is now regular and the fever subdued. His gums and throat are affected by the mercury. Evacuations more natural, and of a less intolerable odour. Complexion rather clearer. Repeat the medicine, but in smaller doses, and omit the ointment.

Fifth day. To day the purging is less frequent, and he only complains of soreness of the bowels with occasional griping pains. Discontinue the calomel, his mouth being more affected, and take the draughts as prescribed, three times a day. Tinct. opii gtts. xxx. conf. aromat. ʒj. ol. menth. ppt. gtt. ij. aqua ad ℥ij. M.

Sixth day. The medicine yesterday promoted a gentle diaphoresis and the pain of the bowels abated. Has had two motions the last twenty-four hours of the natural consistence and colour. An absorbent draught with tinct. opii gtt. xx was given last night. Has now no complaint whatever. To continue the medicine a day or two longer.

What analogy or connection there may subsist in the constitution of fevers of the intermittent and remittent type and the dysentery, it is difficult to pronounce; they, however, change into each other with camelion-like facility, exhibiting the symptoms of each in almost regular succession. Can it be the influence of *bile?* What principally leads me to the conclusion, that superabundance of bile has nothing to do with the dysentery is, that I never myself could observe the least reason for the supposition. The disease is, no doubt, in many instances, owing to obstruction, or otherwise a stimulus, which excites an inverted motion of the parts concerned in its production. But whatever there may be wrong in the hepatic system, it is certain that little or no bile passes by the intestines. This subject affords an extensive field for investigation; and what applies to fevers, generally denominated *bilious*, will be treated of in another place.

[ To be continued. ]

